# A Solid State Zwitterionic Plastic Crystal With High Static Dielectric Constant

**DOI:** 10.1002/adma.202517774

**Published:** 2026-01-16

**Authors:** Zitan Huang, Yifan Liu, Tiago Outerelo Corvo, Ain Uddin, Michelle L. Lehmann, Tomonori Saito, Valentino R. Cooper, Ralph H. Colby

**Affiliations:** 1 Department of Materials Science and Engineering, The Pennsylvania State University, University Park, Pennsylvania, United States; 2 Materials Sciences and Technology Division, Oak Ridge National Laboratory, Oak Ridge, Tennessee, United States; 3 Chemical Sciences Division, Oak Ridge National Laboratory, Oak Ridge, Tennessee, United States

**Keywords:** dielectric relaxation, long-range order, rotational degree of freedom

## Abstract

Solid materials with a high dielectric constant have a wide range of applications in the energy storage field. In this research, an imidazolium-based zwitterion is designed, synthesized, and confirmed to have a plastic crystal phase based on the following experimental and computational evidence: (i) the presence of long-range order with weak intermolecular forces and competing attractive-repulsive interactions along different crystallographic directions; (ii) the observation of more than one endotherm on heating including a solid-solid phase transition at T_s-s_ = −26 °C and melting of the plastic crystal at T_m_ = 72°C; (iii) a low entropy of fusion at melting (2.1 JK^−1^mol^−1^); (iv) a strongly anisotropic morphology; (v) relatively fast dynamics originating from short-range degrees of freedom. Furthermore, it exhibits a very high dielectric constant in the plastic crystal solid state (147 at −10°C and 103 at 70°C) due to the rotational degrees of freedom of plastic crystals that arise from weak net intermolecular interactions of zwitterions due to only having two carbons between the anion and cation. This material conveniently remains in the plastic crystal phase within 50 K of ambient temperature. This discovery opens new opportunities in the search for solid-state high dielectric constant materials.

## 1 | Introduction

The search for materials with a high static dielectric constant (*ε*_s_) has been an important direction in research, as these materials have many applications, such as actuators [1], solid-state batteries [2], and transistors [3]. One of the most promising strategies for achieving materials with high *ε*_s_ is zwitterions, in which cations and anions are covalently bonded, thereby creating highly polar molecules with a high static dielectric constant [4]. It is worth noting, however, that high static dielectric constants are typically achieved in the liquid state since mobile dipoles are required, consequently limiting their range of applications.

Plastic crystals (PCs) are materials with both long-range order (crystalline) and short-range disorder (rotational or reorientational degrees of freedom) contributing to the mechanical plasticity of the material as well as the mobility of dipoles [5]. Since their discovery in the 1960s, most reported PCs are small, nonionic molecules, such as succinonitrile, cyclohexanol, and cyclooctanol, which are termed molecular PCs [6–8]. These materials also showed a considerably high dielectric constant (around 50 for the succinonitrile PC) [9]. The dynamics of these materials have been extensively studied, particularly in the context of solid-state electrolytes for batteries, by adding lithium salt [10–12]. In addition to molecular PCs, organic ionic compounds were first reported to exhibit plastic crystal behavior in the late 1980s and early 1990s by Nakamura and coworkers [13, 14], referred to as organic ionic plastic crystals (OIPCs).

As of today, a variety of OIPCs with different cation-anion combinations have been reported [15]. They are widely used as solid-state electrolytes for batteries due to their high ionic conductivity originating from their ionic nature, and relatively good mechanical properties stemming from their long-range order [16, 17]. For example, Pringle and coworkers developed a series of alkyl ammonium-based OIPCs that achieved high conductivity upon the addition of lithium salt [18]. Although there are few reports of the static dielectric constant of OIPCs, it is expected that they are similar to ionic liquids (ILs) (since ILs can be viewed as the molten counterpart of OIPCs), which typically have a static dielectric constant of around 20 at room temperature [19]. Such a low dielectric constant does not match the emerging need for highly polarizable materials in the energy storage field.

**FIGURE 1 |:**
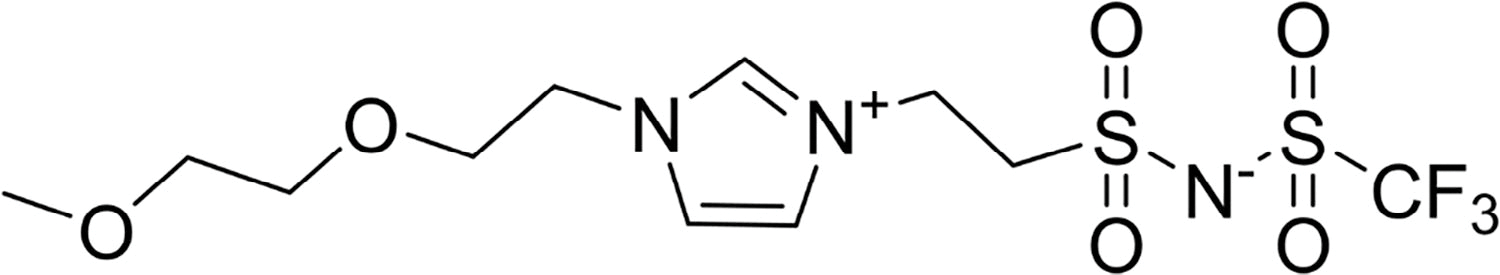
Chemical structure of the 2EOImTSA zwitterion.

An alternative strategy for OIPCs includes zwitterionic PCs, promising candidates for solid-state high static dielectric constant materials. The crystalline nature of PCs ensures that the materials are in a solid state with some mechanical integrity, while the short-range disorder of PCs facilitates the mobility of dipoles, thus promoting a high static dielectric constant. The initial discovery of such material was recently reported by Pringle et al., [20–22]. In this paper, zwitterionic plastic crystal (2-(1-(2-(2-methoxyethoxy)ethyl)-1H-imidazol-3-ium-3-yl)ethyl)sulfonyl)((trifluoromethyl)sulfonyl)amide (2EOImTSA, chemical structure shown in Figure 1) was designed. We hypothesized that having a short distance between cation and anion in the zwitterion would exhibit PC characteristics. The crystal structure of the plastic crystal phase of 2EOImTSA was investigated by both powder and single crystal X-ray diffraction (XRD). Density functional theory (DFT) simulations confirmed that there were very weak net intermolecular forces from competing attractive-repulsive interactions along different crystallographic directions, thus justifying the origin of a PC phase. The larger-scale morphology using atomic force microscopy (AFM) indicated a strongly anisotropic morphology. Differential scanning calorimetry (DSC) and rheology measurements clearly show the existence of a solid-solid state transition process before melting. The short-range rotational dynamics were studied using a combination of broadband dielectric spectroscopy (BDS) and solid-state nuclear magnetic resonance (NMR), which provided evidence for the existence of short-range disorder in this material. The discovery of this PC zwitterion opens new opportunities for designing and discovering new solid materials with a high dielectric constant.

## 2 | Results and Discussion

### 2.1 | Synthesis of 2EOImTSA

**SCHEME 1 |:**
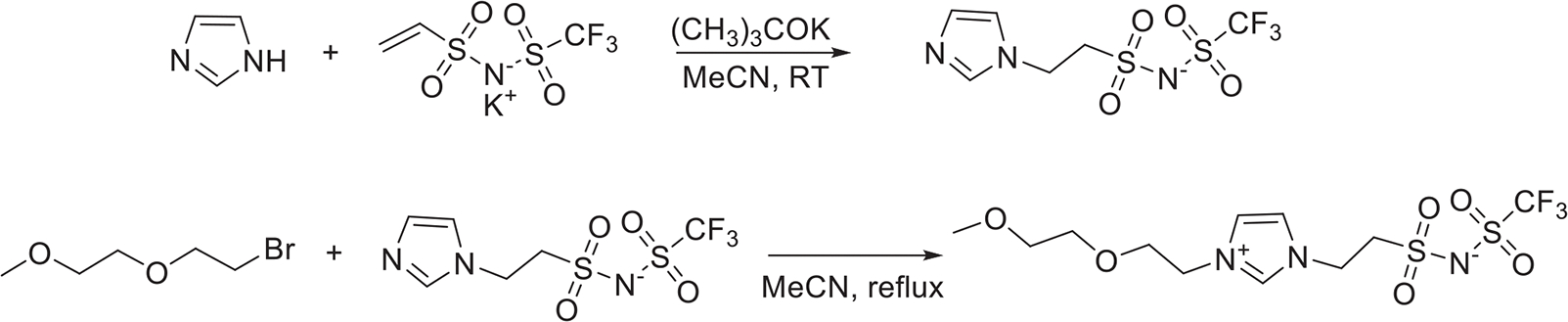
Synthesis pathway of ((2-(1-(2-(2-methoxyethoxy)ethyl)-1H-imidazol-3-ium-3-yl)ethyl)sulfonyl)((trifluoromethyl)sulfonyl)amide, (2EOImTSA).

2EOImTSA was synthesized by reacting imidazole with vinyl trifluoromethanesulfonimide (VTFSI), followed by reacting with 1-bromo-2-(2-methoxyethoxy)ethane (Details are included in the METHODS section (Scheme 1.), Figures S1 and S2).

### 2.2 | Characterizing the Plastic Crystal Phase in 2EOImTSA

**FIGURE 2 |:**
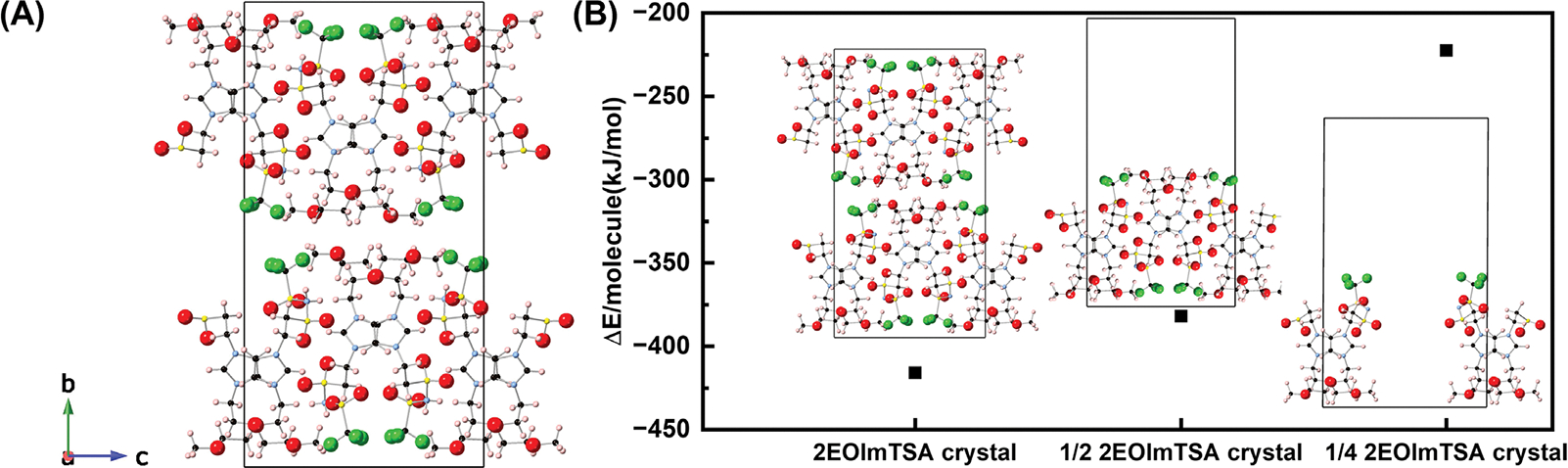
(A) Crystal structure of 2EOImTSA, viewed along the a-axis obtained from single-crystal XRD at room temperature. The definition of the *a*, *b*, and *c* axes is described in the legend located in the bottom-left corner. (B) Cohesive energies from DFT calculations for 2EOImTSA systems in different configurations to understand the interactions in the 2EOImTSA crystal using eq. 1. For the 2EOImTSA crystal, *n* = 8; 1/2 EOImTSA crystal for ½ 2EOImTSA crystal, *n* = 4; for ¼ 2EOImTSA crystal, *n* = 2. F-green, S-yellow, N-blue, C-black, O-red, H-pink.

**FIGURE 3 |:**
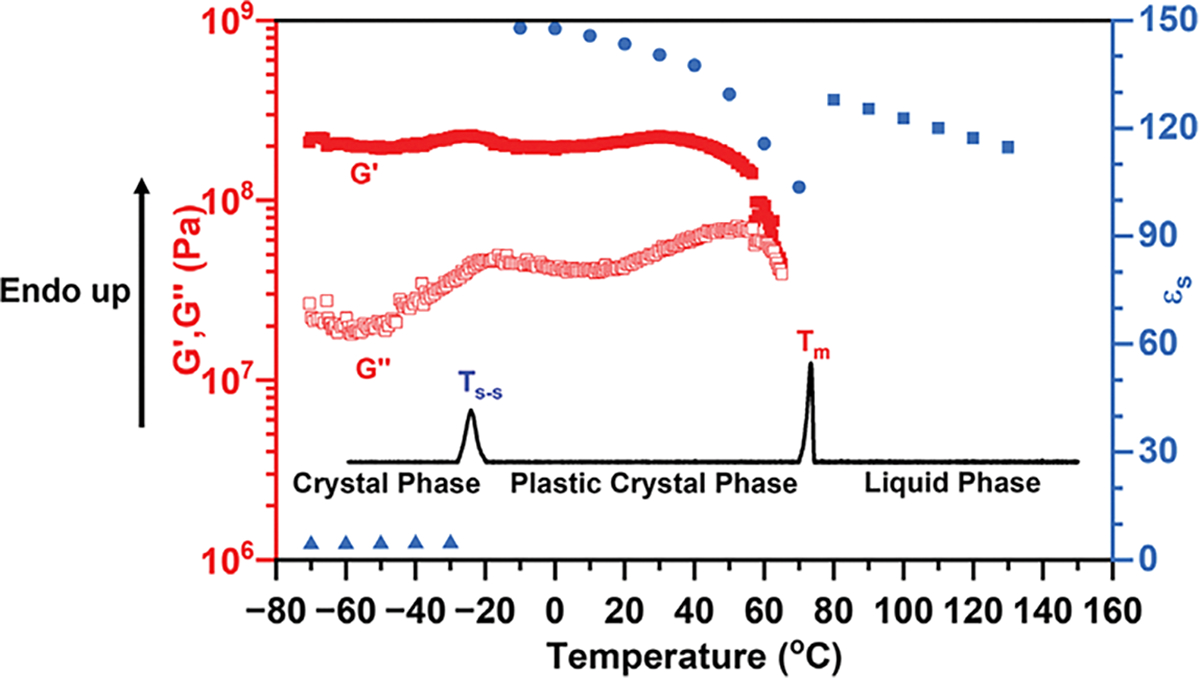
Temperature-dependent thermal, mechanical (*G′, G″)*, and dielectric properties of 2EOImTSA. DSC thermogram (solid black line) of 2EOImTSA with a heating rate of 1K/min, with enthalpy changes for the solid-solid phase transition ΔH_S-S_ = 1.59 J/g, and the melting process ΔH_m_ = 1.80 J/g. Oscillatory temperature sweep of 2EOImTSA with a heating rate of 2K/min at 1 rad/s using a 3 mm aluminum parallel plate geometry. Static dielectric constant (*ε*_s_, blue data points) as a function of temperature.

As summarized by previous literature, PCs (i) show one or more solid-solid phase transitions prior to melting; (ii) have a low entropy of fusion ΔS_f_ ≤20 JK^−1^mol^−1^, due to the short-range disorder, and (iii) show strong long-range order (a crystalline structure) [6, 15, 17, 23]. To confirm that 2EOImTSA has a PC phase, its structural, thermal, and dielectric properties were characterized using XRD, DFT (Figure 2), DSC, BDS, and rheology (Figure 3).

The crystal structure of 2EOImTSA was obtained from single-crystal (SC) XRD measurements. A detailed experimental procedure for single crystal growth and data analysis is included in the METHODS section, as well as supporting information (Figure S3). Coincidentally, the space group of this crystal structure (P 21/C) is the same as the only other reported PC zwitterion by Pringle and coworkers [20]. This SC-XRD result, together with the powder XRD spectra (Figure S4), confirms the existence of long-range order of 2EOImTSA, which corresponds to characteristic (iii) of the plastic crystal mentioned previously.

To elucidate the intermolecular interactions underlying plastic crystal formation in 2EOImTSA, cohesive energies were calculated from DFT-optimized configurations. The cohesive energy was determined using the equation:

(1) $$\Delta E/molecule=\frac{\left(E_{\mathrm{total}}\;-\;nE_{\mathrm{molecules}}\right)}{n}$$

where $E_{\mathrm{total}}$ is the total energy of the optimized molecular assembly, $E_{\mathrm{molecules}}$ is the energy of a single isolated molecule, and n is the number of molecules in the assembled system. The details of the DFT calculations can be found in the METHODS section. The analysis compared several structural arrangements, as shown in Figure 2B: (i) the complete SC-XRD crystal structure containing 8 molecules per unit cell; (ii) a half-crystal system (½ 2EOImTSA) with 4 molecules per unit cell, where half the system along the *b*-axis was replaced with vacuum; (iii) a quarter-crystal system (¼ 2EOImTSA) with 2 molecules per unit cell, created by additionally removing antiparallel ion pairs (+−,+−) from the 1/2 EOImTSA crystal;

The cohesive energy calculations revealed distinct stability patterns across these configurations. The dimer 2EOImTSA ++,−− arrangement exhibited the weakest cohesive energy (−117.63 kJ/mol) with a longer distance between the two molecules, indicating repulsive interactions between like-charged species (Figure S9). In contrast, the dimer 2EOImTSA +−,+− configuration demonstrated favorable stability with a cohesive energy of −161.41 kJ/mol (Figure 6A). Notably, the ¼ 2EOImTSA crystal, despite containing the 2EOImTSA ++,−− arrangement along the a-axis, showed enhanced stability compared to the isolated dimer in vacuum, suggesting cooperative effects in the extended structure along the *a*-axis. The ½ 2EOImTSA crystal exhibited substantially stronger intermolecular interactions (cohesive energy: −382.01 kJ/mol) due to additional 2EOImTSA +−,+− pairs along the *c*-axis. The complete 2EOImTSA crystal structure achieved the highest stability with a cohesive energy of −416.05 kJ/mol.

These results suggest that crystal stability is primarily driven by strong anion–cation attractive interactions, which contribute to the overall thermodynamic favorability of the assembled structure. Conversely, the observed plastic crystal behavior arises from directional weakening of binding due to repulsive interactions between like-charged species.

In other words, the plastic crystal formation in 2EOImTSA results from a delicate balance between attractive anion-cation interactions that stabilize the crystal structure and repulsive-like charge interactions that facilitate molecular mobility. This combination of weak intermolecular forces and competing attractive-repulsive interactions along different crystallographic directions constitutes the fundamental mechanism for plastic crystal behavior in these assembled zwitterionic systems.

**FIGURE 4 |:**
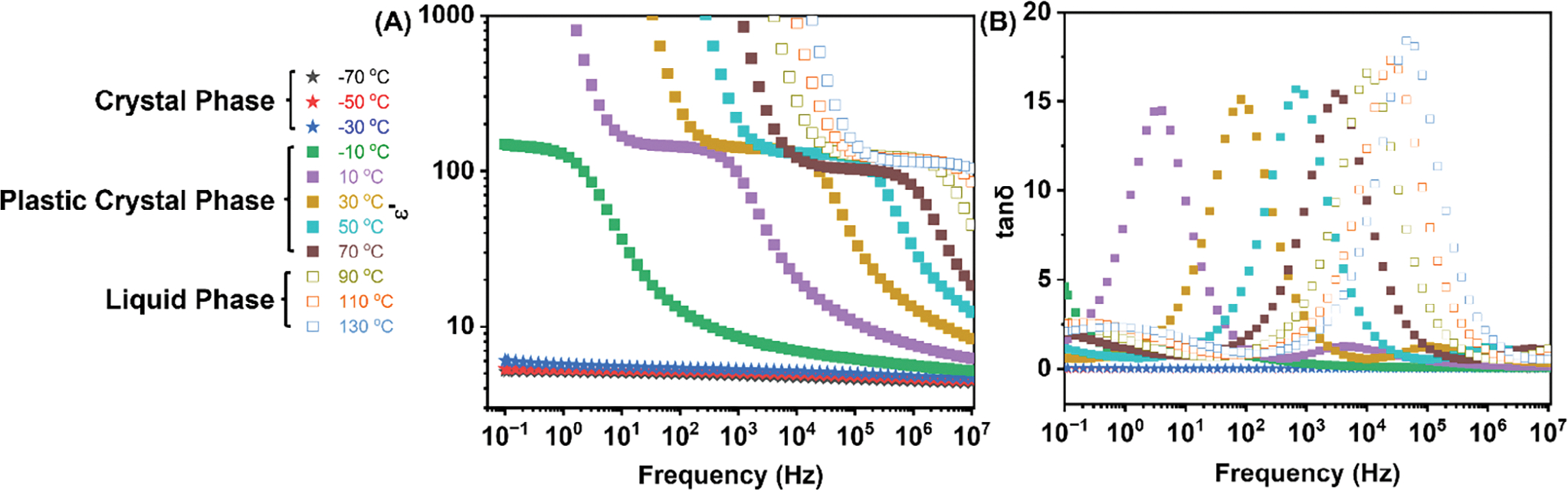
(A) Permittivity (*ε*′) as a function of frequency at temperatures from −70°C to 130°C; (B) dielectric loss (tan*δ*=ɛ″/ɛ′) as a function of frequency at temperatures from −70°C to 130°C.

As shown in Figure 3, at temperatures below T_S-S_, the static dielectric constant (*ε*_s_) remains nearly constant and low, which is expected for regular crystals because of the lack of dipole mobility. At the same time, the storage modulus (G′) (roughly similar to the elastic modulus (E) of another organic plastic crystal reported by Reddy and coworkers [24]) is larger than the loss modulus (G″), which confirms that the material is a solid in this temperature range. When the temperature increases to T_S-S_, an endothermic peak is shown on the DSC thermogram. This solid-solid phase transition is also revealed by a peak in G″ on the oscillatory shear data. Such observation aligns with requirement (i) of PCs, as the temperature is further increased above T_S-S_, the static dielectric constant increases dramatically. This indicates that, despite the material remaining solid, individual dipoles become mobile, as expected from the rotational freedom of PCs. Qualitatively similar dielectric properties were reported by Lunkenheimer and coworkers [11, 12, 25] for non-ionic polar molecular PCs, with a far lower dielectric constant [9]. In the plastic crystal phase, the static dielectric constant (*ε*_s_) (obtained from the plateau value in Figure 4A) decreases with increasing temperature, due to the thermal randomization of dipoles. A very high static dielectric constant (147 at −10°C, and 103 at 70°C before melting) was obtained in the plastic crystal phase of our zwitterion, 2EOImTSA. The relatively high loss modulus in the PC phase compared to the lower temperature true crystal phase suggests a larger dissipation, hence some degree of plasticity of 2EOImTSA in this temperature range. As the temperature increases further, another endothermic peak is observed on the DSC thermogram. Combined with modulus data, we can assign this peak to a melting process. The entropy of fusion for the melting process is calculated to be 2.1 JK^−1^mol^−1^, a rather low value, roughly similar to phosphonium-based OIPC (5 JK^−1^mol^−1^) [26], and far below Timmermans′ proposed upper bound for a PC phase of 20 JK^−1^mol^−1^ [6]. This result aligns with character (ii) of PCs. Therefore, 2EOImTSA fits with the three main characteristics of PCs, confirming that 2EOImTSA is a PC. As the temperature increases above the melting point, the sample becomes a pourable liquid, resulting in an abrupt increase in static dielectric constant, signaling the increased degree of freedom of the dipoles in the liquid state. The static dielectric constant then follows a typical temperature dependence of any polar liquid after melting. In summary, there are three phases observed on heating: a crystal phase below T_s-s_ = −26°C, a PC phase, and a liquid phase above T_m_ = 72°C. The PC phase covers a wide temperature range (from −26°C to 72°C), which includes room temperature.

The dielectric response of the 2EOImTSA is reviewed in Figure 4. As is shown in Figure 4B, after entering the plastic crystal phase, the dielectric loss shows a significant increase and shows a peak. Such an increase in dielectric loss and also the formation of the peak is due to the strong dipolar relaxation strength in the plastic crystal phase. Such a high dielectric loss might prevent this material from applications at certain frequency ranges. More research is needed to design zwitterionic PCs with a lower dielectric loss in the future.

### 2.3 | Morphology of 2EOImTSA

**FIGURE 5 |:**
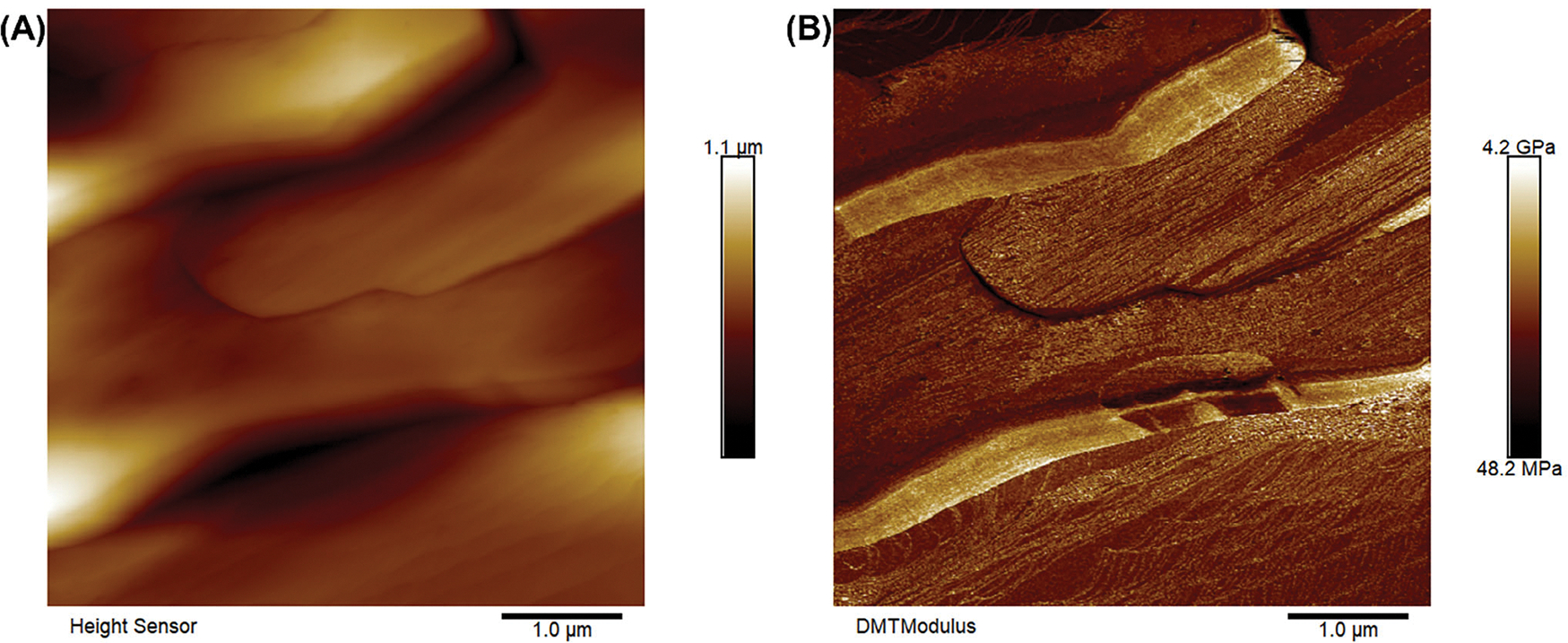
(A) AFM topology image of 2EOImTSA at room temperature using peak force tapping mode. (B) AFM modulus image of 2EOImTSA at room temperature using peak force tapping mode. The spacing between the lines is 68 nm.

To further understand the origin of the mechanical plasticity of 2EOImTSA, the morphology of the material at room temperature was characterized using AFM. Both the AFM topology image (Figure 5A) and modulus image (Figure 5B) show that 2EOImTSA has a highly anisotropic morphology. Such morphology further leads to the mechanical plasticity (evidenced by the relatively high G″ in the plastic crystal phase, Figure 3), and a similar morphology was also observed by Pringle and coworkers [20] for their plastic crystal zwitterion. Additionally, the modulus measured by AFM (Figure 5B) agrees roughly well with the rheological measurement results (Figure 3). The storage modulus (G′) measured in oscillatory shear at 25°C is 0.22 GPa, similar to the modulus measured using AFM (Figure 5B). AFM images in other spots of the sample (Figure S5) can further prove the anisotropic morphology of 2EOImTSA.

### 2.4 | Dynamics of 2EOImTSA

**FIGURE 6 |:**
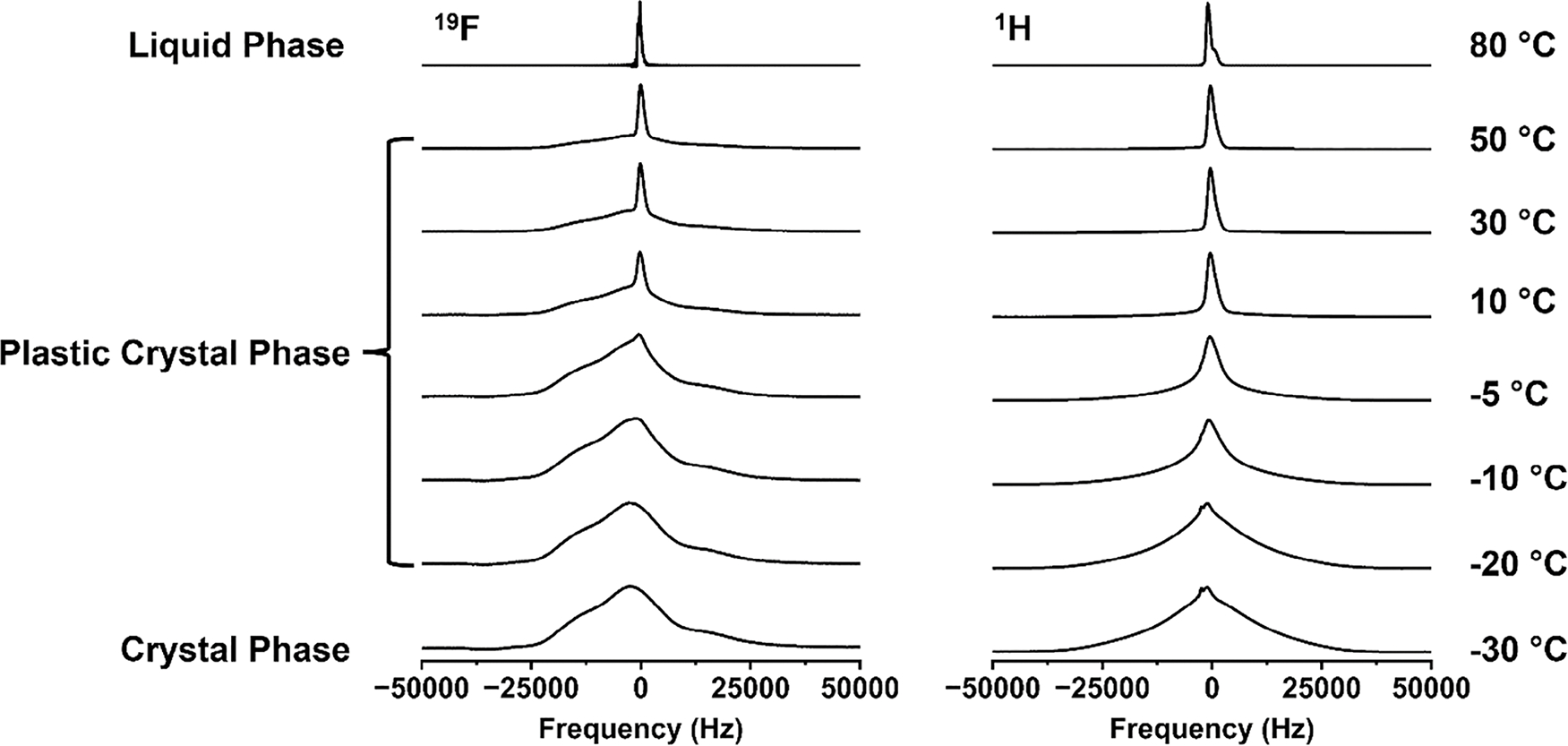
Solid state ^1^H and ^19^F NMR spectra of 2EOImTSA at temperatures from −30°C to 80°C. ^1^H (right) and ^19^F NMR (left) spectra peak shape as a function of temperature in three different phases.

From the previous results, it is proven that 2EOImTSA has a plastic crystal phase, and the existence of a rotational degree of freedom leads to a high dielectric constant in its solid state. To further qualitatively characterize the short-range rotational dynamics of 2EOImTSA, solid-state NMR spectra were obtained. The dynamics of 2EOImTSA can be captured by the shape of the peak in the solid-state NMR spectra, reported in Figure 6. At 80°C (top of Figure 6) both ^1^H and ^19^F spectra are narrow, as expected from any liquid. Both spectra broaden as the temperature is lowered, indicating that molecular motion becomes more restricted, commensurate with the emergence of long-range order. While the ^1^H NMR spectra broaden and maintain symmetry, the ^19^F spectra, reflecting the dynamics of the TSA anion of 2EOImTSA, show a sharp peak superimposed on a very broad base in the PC phase. This is a critical characteristic of PCs [16, 18, 20, 27], originating from short-range disorder (whose origin was explained by DFT simulations in Figure 2), although not fully understood. In the PC phase, it is apparent that the sharper component becomes less dominant as temperature decreases, and this shows the gradual decrease of the molecular mobility of the zwitterion in the PC phase. Below roughly 0°C, these two merge into a broad peak that persists in the true crystal phase (at −30°C). An abrupt narrowing of the peak is observed between −10°C and −5°C for ^19^F NMR spectra, while a similar abrupt narrowing was observed between −20°C and −15°C for ^1^H NMR spectra (Figure S7). Apparently, the proton-containing moiety of 2EOImTSA (cation and ether oxygen side chain) gained some mobility at a lower temperature than the fluorine-containing TSA anion.

As discussed briefly in the Introduction, developing solid-state zwitterions with a high dielectric constant has been a main research focus. Prominent approaches have focused on the development of cross-linked or polymeric zwitterions to maintain mechanical integrity [4]. Polyzwitterions with relatively high static dielectric constant and relatively good mechanical integrity were reported in several of our recent publications [28, 29]. However, most polyzwitterions are amorphous, not taking advantage of the potential formation of crystal structures, and relatively complex syntheses are typically needed for polyzwitterions, with some limitation in achieving high zwitterion-content polymers. Compared with polyzwitterions, little attention has been paid to developing PC zwitterions, which should be ideal candidates for solid-state, high dielectric constant materials.

Currently, the most heavily investigated and widely used high-K materials are polymers [30] and ceramics [31, 32]. Typically, polymers show a modest dielectric constant of 2–12 at room temperature, and the benchmark polymers are ferroelectric poly(vinylidene fluoride)-based materials which can achieve a dielectric constant as high as 20 at room temperature [33–35]. For some rare cases, the dielectric constant of a solid polymerceramics composite can reach over 100 with a large amount of ceramic doping [36]. Such a dielectric constant is lower than that of the 2EOImTSA, and the processability of 2EOImTSA should be facilitated by the fact that it is a liquid above T_m_ = 72°C. However, compared with polymers, the mechanical properties of 2EOImTSA are worse and further improvements are needed. On the other hand, ceramics generally show a much higher dielectric constant (typically larger than 100)[37, 38], and the dielectric constant of most of the ceramics is at least on par with or even higher than the dielectric constant of 2EOImTSA in its solid state. However, the high processing temperature of ceramics (typically above 1000°C) and mechanical brittleness greatly limit their applications compared with those of PC zwitterions [39]. Therefore, in general, the PC zwitterion 2EOImTSA shows unique competitive strength compared with both polymer and ceramic solid dielectric materials.

**FIGURE 7 |:**
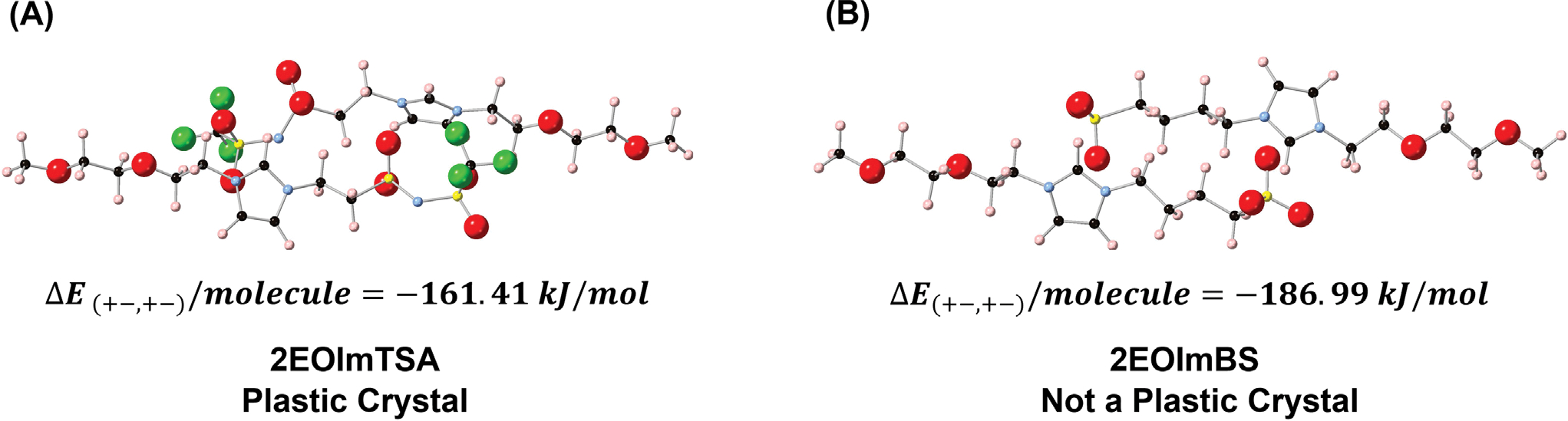
The review of cohesive energy and optimized structure. F-green, S-yellow, N-blue, C-black, O-red, H-pink. (A) Optimized structure using Quantum ESPRESSO and cohesive energy of 2EOImTSA calculated using Equation 1. with *n* = 2. (B) Optimized structure using Quantum ESPRESSO and cohesive energy of 2EOImBS calculated using Equation 1. with *n* = 2.

Although there are few PC zwitterions reported, we believe that there are many other PC zwitterions that remain undiscovered. For example, Ohno and coworkers observed one additional solid-solid state transition before melting for several zwitterions, i.e., potentially PC zwitterions [40]. In this vein, an appropriate screening criterion is needed for designing and discovering new PC zwitterions. To create them, we first need to understand what is special about 2EOImTSA that makes it a PC. As is well known for molecular PCs, weak interactions between molecules underpin the formation of PCs [41]. Here, density functional theory (DFT) can be a valuable tool for calculating the cohesive energy between potential PC molecules, which can then be used as a descriptor for screening PCs. As discussed, we calculated the cohesive energy between 2EOImTSA dimers (Figure 7A). As a comparison, the cohesive energy for the 4-(1-(2-(2-methoxyethoxy)ethyl)-1H-imidazol-3-ium-3-yl)butane-1-sulfonate (2EOImBS) dimer (a zwitterion with a similar chemical structure and proven not to show any PC phase) was also calculated and reviewed in Figure 7B. It is apparent that the cohesive energy between 2EOImTSA (−161.41 kJ/mol) is 14% smaller than that of 2EOImBS (−186.99 kJ/mol). These results suggest that the intermolecular cohesive energy might be a suitable screening criterion for PC zwitterions. Together with emerging data science and machine learning tools, such a descriptor can make the discovery of new PCs much less time and resource-intensive.

## 3 | Conclusion

In this paper, a zwitterionic plastic crystal (2EOImTSA) was synthesized. The existence of a plastic crystal phase was confirmed by matching the experimental results with the three criteria of plastic crystals. Additionally, the origin of short-range disorder and long-range order characteristic of the plastic crystal phase was investigated, and it was proven to be due to weak intermolecular forces and competing attractive-repulsive interactions along different crystallographic directions. Due to the dipole moment mobility originating from the short-range disorder, a high dielectric constant in its solid plastic crystal phase (147 at −10°C and 103 at 70°C) was achieved. This material conveniently remains in the plastic crystal phase, hence with a high dielectric constant, within 50 K of ambient temperature, ideal for solid-state electrolyte applications. Future investigation confirms that cohesive energy is shown to be an insightful screening criterion for future plastic crystal zwitterion research. Future investigations to target potential applications include mixing the zwitterion with a lithium salt to make high dielectric constant solid-state electrolytes and highly polarizable solids, such as actuators and supercapacitors. For initial trials, we mixed 8 wt.% LiTFSI with 2EOImTSA. The mixture was a liquid, and a reasonable conductivity of 3.4 × 10^−4^ S/cm at 130°C was obtained. In contrast, Pringle was able to add 6.07 wt.% LiTFSI (10 mol%) and maintain the plastic crystal state for the zwitterion [20]. We hope that this work motivates future investigations to target potential applications of zwitterionic plastic crystals.

## 4 | Methods

### 4.1 | Materials

Imidazole (99%, Sigma–Aldrich), 1-bromo-2-(2-methoxyethoxy)ethane (90%, Ambeed), and potassium *tert*-butoxide (98%, Sigma–Aldrich) were used as received. Potassium vinyl trifluoromethanesulfonimide (VTFSI K) was synthesized as previously reported [42].(Scheme 1)

### 4.2 | 2EOImTSA Synthesis

Imidazole (1.5 g, 21.6 mmol), VTFSI K (4.0 g, 14.4 mmol), and potassium *tert*-butoxide (21.6 mmol) were added to anhydrous acetonitrile (MeCN, 12 mL) under argon. The solution was mixed overnight at room temperature, then the reaction mixture was poured into dichloromethane (DCM). The solid product (imidazole-TFSI) was collected by vacuum filtration and washed with DCM to yield a white powder (4.5 g, isolated yield 82%).^1^H NMR (300 MHz, DMSO-d_6_, *δ*): 3.44 (t, 2H), 4.31 (t, 2H), 6.87 (s, 1H), 7.21 (s, 1H), 7.65 (s, 1H).

Imidazole-TFSI (4 g, 11.6 mmol) and 1-bromo-2-(2-methoxyethoxy)ethane (5.3 g, 28.9 mmol) were added to anhydrous MeCN (16 mL) in a pressure vessel under argon. The solution was reacted at 85°C overnight, before cooling to room temperature and centrifuged to remove insoluble KBr. The solvent was removed, and the resulting solids were dissolved in DCM and centrifuged to further remove any insoluble by-products. The product, 2EOImTSA, was recrystallized from DCM 2x to yield a white crystalline solid (3.8 g, isolated yield 41%). ^1^H NMR (300 MHz, DMSO-d_6_, *δ*): 3.23 (s, 3H), 3.42 (dd, 2H), 3.54 (dd, 2H), 3.59 (t, 2H), 3.75 (t, 2H), 4.33 (t, 2H), 4.57 (t, 2H), 7.71 (s, 1H), 7.82 (s, 1H), 9.17 (s, 1H).

### 4.3 | Broadband Dielectric Spectroscopy (BDS)

2EOImTSA was dissolved in acetonitrile, and the solution was cast on a freshly polished brass bottom electrode equipped with four 0.1 mm glass fiber spacers used to control and maintain the thickness of the sample. The electrode was first dried in a convection oven at 70°C overnight to evaporate most of the solvent. The electrode was then covered with a 10 mm diameter top brass electrode and dried under vacuum at 70°C for 36 h to remove the remaining solvent and moisture. The whole sandwich cell was clamped onto a BDS 1200 sample cell (Novocontrol Technologies) and inserted into a BDS 1100 cryostat for temperature control in a dry nitrogen environment. The measurement was then performed on an Alpha High-Resolution Broadband Dielectric/Impedance Spectrometer (Novocontrol Technologies) with 0.1 V excitation and no DC bias. At each temperature, the data were obtained from an isothermal frequency sweep in the frequency range of 0.1–10^7^ Hz.

### 4.4 | Differential Scanning Calorimetry (DSC)

DSC measurement was conducted on a DSC 8500 (PerkinElmer) instrument. The sample was loaded into an aluminum pan, which was then sealed. The pan was further dried under vacuum at 70°C for 24 h to remove moisture picked up during the sample preparation. The pan was then quickly loaded into the instrument together with an empty reference pan. The sample was heated up from −60°C to 160°C with a heating rate of 1 K/min. The baseline was subtracted using Origin software.

### 4.5 | Solid-State NMR

The sample was dried under vacuum at 70°C for 36 h and was then stored inside a glove box. It was then packed into a 5 mm glass NMR tube inside the glove box, and the cap was sealed with parafilm. Both ^1^H NMR and ^19^F NMR were performed on an AVANCE NanoBay NEO-400 instrument (Bruker) at various temperatures. The sample was kept at each temperature for 20 min before running the measurement.

### 4.6 | Linear Viscoelastic Rheological Response

Small amplitude oscillatory shear (SAOS) temperature sweep was conducted on an ARES-G2 (TA Instruments) strain-controlled rheometer using a 3 mm aluminum parallel plate geometry. To make sure the measurements were taken in a linear regime, strain amplitude sweeps were performed at −70°C and 20°C at 1 rad/s. The measurement was performed from −70°C to around 60°C when the sample started melting. During the measurement, a heating rate of 2 K/min, an angular frequency of 1 rad/s, and a strain amplitude of 0.05% were applied.

### 4.7 | X-Ray Diffraction (XRD)

The powder XRD measurement was conducted on a Rigaku X-ray instrument (Rigaku), equipped with a MicroMax-007 HF Cu *Kα* source (*λ* = 1.54Å) and a HyPix-Arc 150° (Rigaku) detector. A powder sample was loaded onto a small nylon loop under an optical microscope and then placed onto a Universal 4-circle kappa goniometer. The signal was detected by a Rigaku HyPix-Arc 150° detector. The temperature was controlled by a Cobra open-flow cooler (Oxford Cryosystems). The measurement was performed at various temperatures. Two measurements were taken at each temperature, with an exposure time of 300 s to make sure the sample reached its equilibrium state at each temperature. Single-crystal XRD measurement was performed on the same instrument as the powder XRD. The single crystal of 2EOImTSA was obtained by recrystallizing it from its MeCN (0.1 g/mL) solution in a freezer at −15°C.

### 4.8 | Atomic Force Microscopy (AFM)

AFM was measured using a Dimension Icon (Bruker) instrument equipped with an RTESPA-150 probe. The sample was prepared by solvent casting a 2EOImTSA solution onto a sample disk. The sample was dried under vacuum and stored in the glove box before measurements. The topology data obtained were flattened using the NanoScope Analysis software. Both topology and modulus images were also exported from NanoScope Analysis software.

### 4.9 | Density Functional Theory (DFT) Calculation

DFT calculations were performed using Quantum ESPRESSO (v7.3.1)[43] to determine cohesive energies for various 2EOImTSA configurations and the 2EOImBS +−,+− system. For non-crystalline 2EOImTSA and 2EOImBS structures, gas-phase simulations were conducted with molecules placed in vacuum boxes maintaining a minimum separation of 16 Å and the Makov-Payne correction to prevent spurious interactions between periodic images. These calculations employed a Γ-point sampling of the Brillouin zone. The computational parameters included a plane-wave kinetic energy cutoff of 100 Ry and a self-consistent field (SCF) convergence criterion of 10^−8^ Ry. GBRV ultrasoft pseudopotentials [44] were utilized within the PBE framework, combined with the vdW-DF-C09 exchange-correlation functional [45] to accurately capture van der Waals interactions. The valence electron configurations used in the pseudopotentials were as follows: hydrogen (H): 1s^1^, carbon (C): [He] 2s_2_2p_2_, nitrogen (N): [He] 2s_2_2p_3_, oxygen (O): [He] 2s_2_2p_4_, fluorine (F): [He] 2s_2_2p_5_, and sulfur (S): [Ne] 3s_2_3p_4_. For the crystalline 2EOImTSA system, variable-cell relaxation calculations were performed using a 2 × 1 × 2 k-point mesh to ensure adequate sampling of the Brillouin zone. The benchmark systems of one molecule started with linear structures similar to the configurations in Figure 7.

[CCDC 2481463contains the supplementary crystallographic data for this paper. These data can be obtained free of charge from The Cambridge Crystallographic Data Centre via www.ccdc.cam.ac.uk/data_request/cif]

## Supplementary Material

Supporting Information

Supporting Information

Additional supporting information can be found online in the Supporting Information section.

**Supporting Information File 1**: adma71922-sup-0001-SuppMat.docx

## Data Availability

BDS, rheology, and DFT calculation data have been deposited at Zenodo a https://doi.org/10.5281/zenodo.16896304 and is publicly available as of the date of publication. Any additional information required to reanalyze the data reported in this paper is available from the lead contact upon request.
