# Supplementary material for: A Solid State Zwitterionic Plastic Crystal With High Static Dielectric Constant
Source: Adv Mater. Author manuscript; Available in PMC 2026 Apr 8. (PMC12921328; doi:10.1002/adma.202517774)
Supplement: Supporting Information [file NIHMS2144783-supplement-Supporting_Information.pdf]

Supporting Information

A Solid State Zwitterionic Plastic Crystal with High Static Dielectric Constant

Zitan Huang[1], Yifan Liu[2], Tiago Outerelo Corvo[1], Ain Uddin[3], Michelle L. Lehmann[3], Tomonori Saito[3], Valentino R. Cooper[2], and Ralph H. Colby[1,*]

[1]Department of Materials Science and Engineering, The Pennsylvania State University, University Park, Pennsylvania 16802, United States.

[2]Materials Sciences and Technology Division, Oak Ridge National Laboratory, Oak Ridge, Tennessee 37831, United States

[3]Chemical Sciences Division, Oak Ridge National Laboratory, Oak Ridge, Tennessee, 37831, United States.

NMR Spectra of imidazole-TFSI and 2EOImTSA

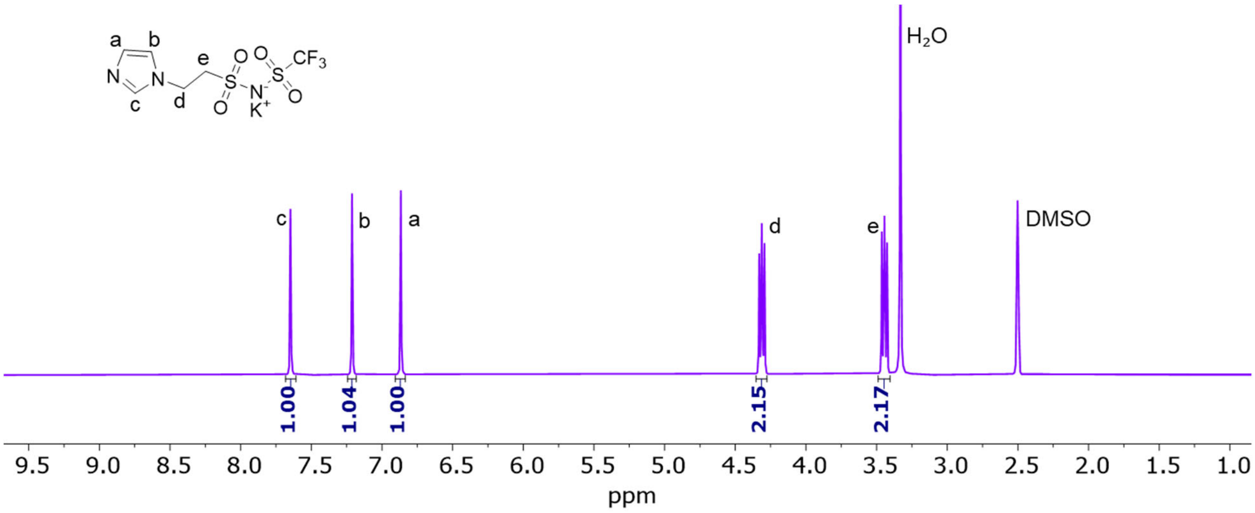

Figure S1. ^{1}H NMR spectra of imidazole-TFSI in dimethyl sulfoxide, DMSO-d6.

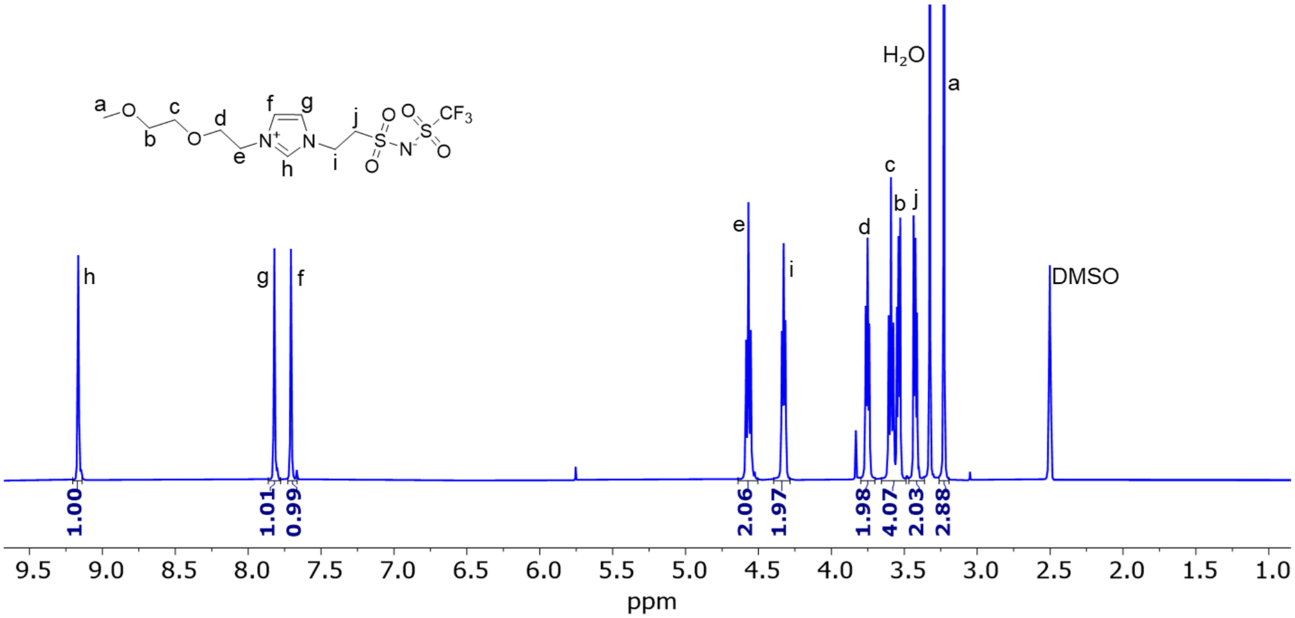

Figure S2. ^{1}H NMR spectra of 2EOImTSA in DMSO-d6.

As shown in Figure S2, ^{1}H NMR spectra prove the successful synthesis of 2EOImTSA. The integrations all approximately align with the associated number of protons for each peak. The integration of ‘c’ and ‘b’ is combined.

Single-Crystal X-Ray Diffraction (SC-XRD) Pattern

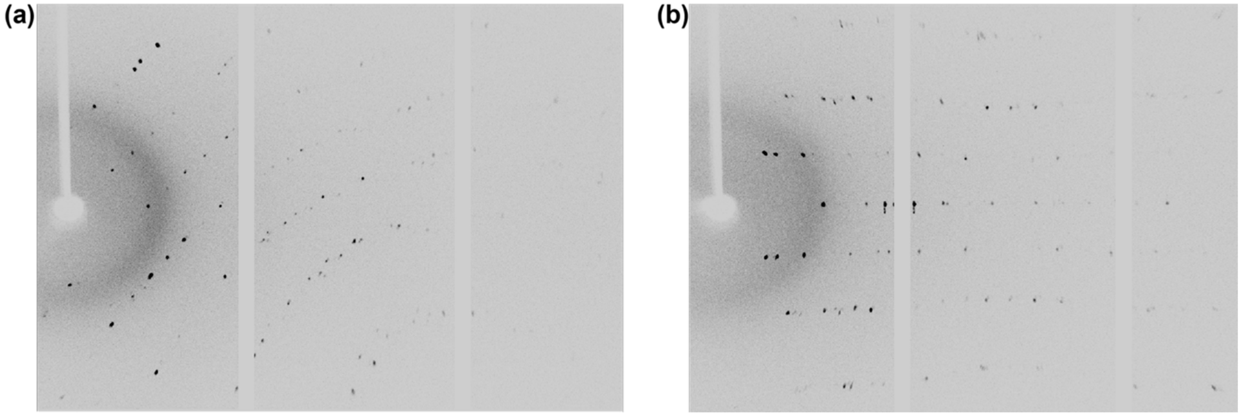

Figure S3. SC-XRD scattering pattern of 2EOImTSA

Figure S3 shows the SC-XRD pattern. Olex2 software was used to obtain the crystal structure (Figure 2 of the main text) using Charge Flipping, which was refined with the SHEXL refinement package using Least Squares minimization.[1-3]

Powder XRD Pattern

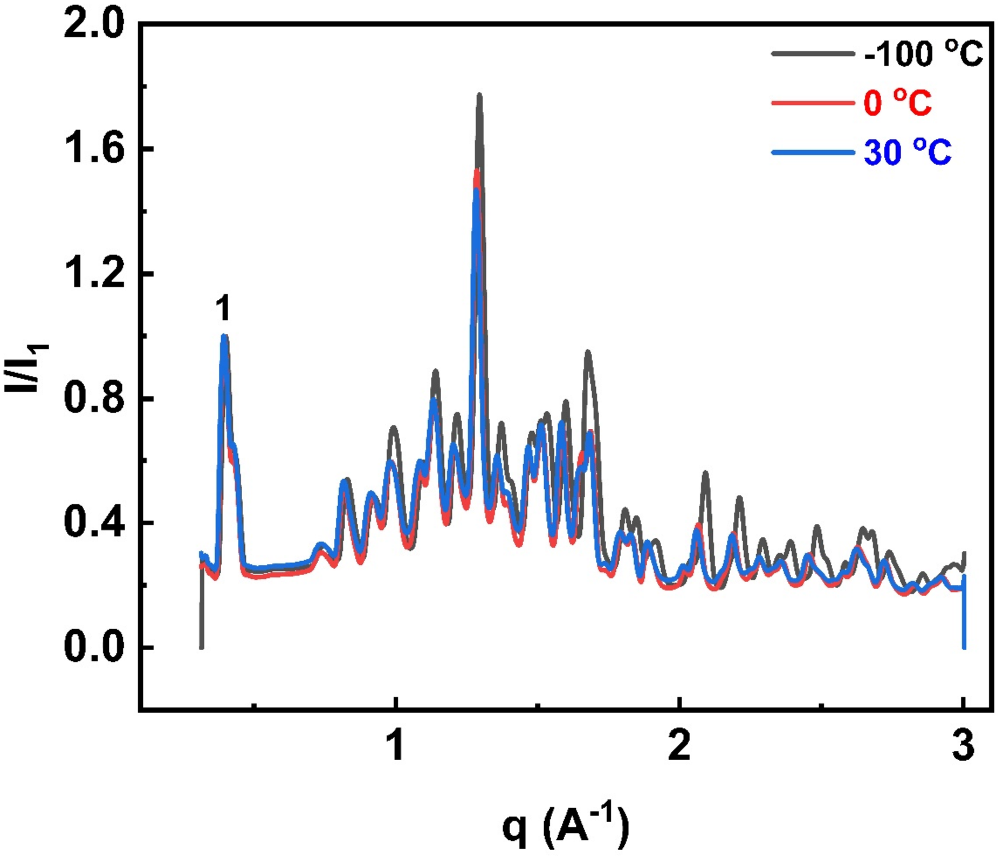

Figure S4. Powder XRD spectra of 2EOImTSA plotted as intensity relative to peak 1 (I/I_1) as a function of scattering vector (q) at -100 °C (true crystal), 0 °C, and 30 °C (both in the plastic crystal phase).

As shown in Figure S4, the XRD spectra of 2EOImTSA show multiple sharp crystalline peaks, as expected for a plastic crystal. Additionally, the low q peak shows nearly no change in terms of peak intensity and position, while the high q peaks broaden as temperature increases. This suggests that the long-range order persists while the short-range order is progressively lost, as is expected from the orientational mobility in the plastic crystal.

Dielectric Properties of 2EOImTSA

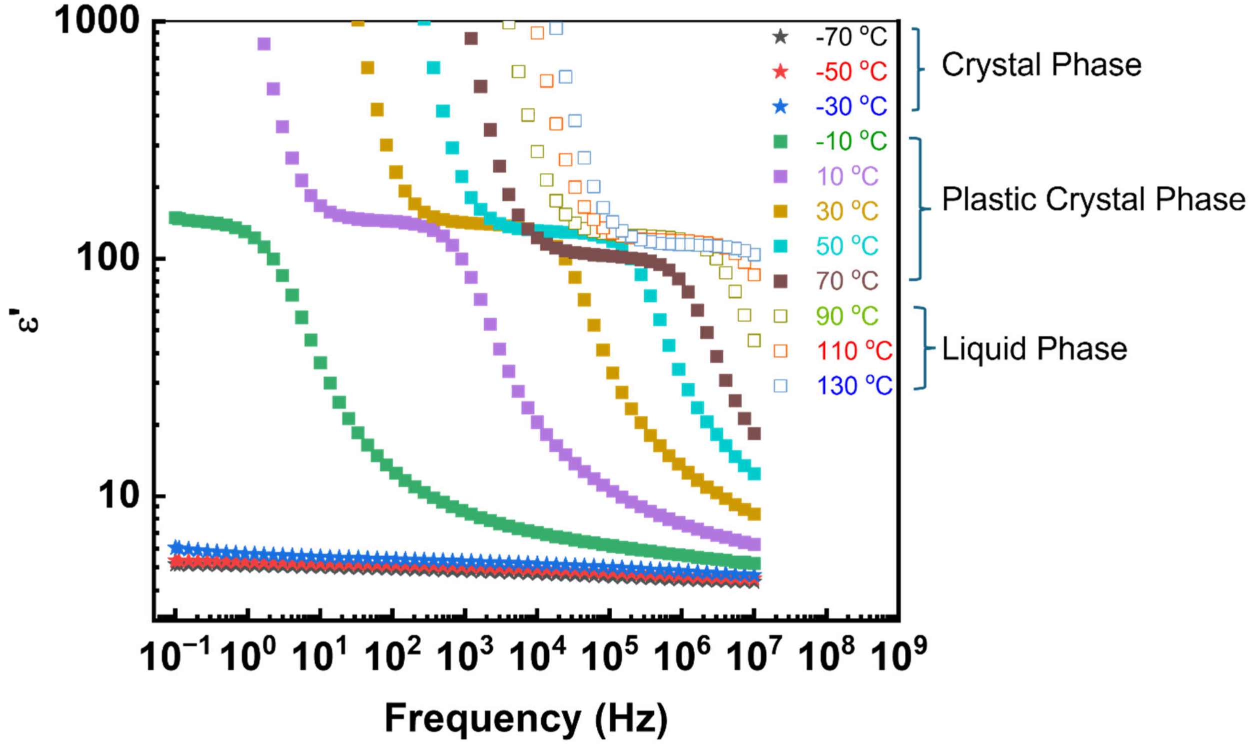

Figure S5. Permittivity (ε') as a function of frequency at temperatures from -70 to 130 °C.

The dielectric properties of 2EOImTSA were probed using BDS as reviewed in the METHODS Section. As shown in Figure S5, the permittivity (ε') remains nearly constant and low (around 4.5) at temperatures below -30 °C in the frequency range of measurements (0.1-10^7 Hz). As the temperature increases, the permittivity increases drastically after entering the plastic crystal phase. The static dielectric constant plotted in Figure 3 is obtained from the plateau value in Figure S5. The strong frequency dependence at frequencies lower than the plateau is from electrode polarization, and the molecules are only partially polarizing at frequencies higher than the plateau.

AFM Images of 2EOImTSA

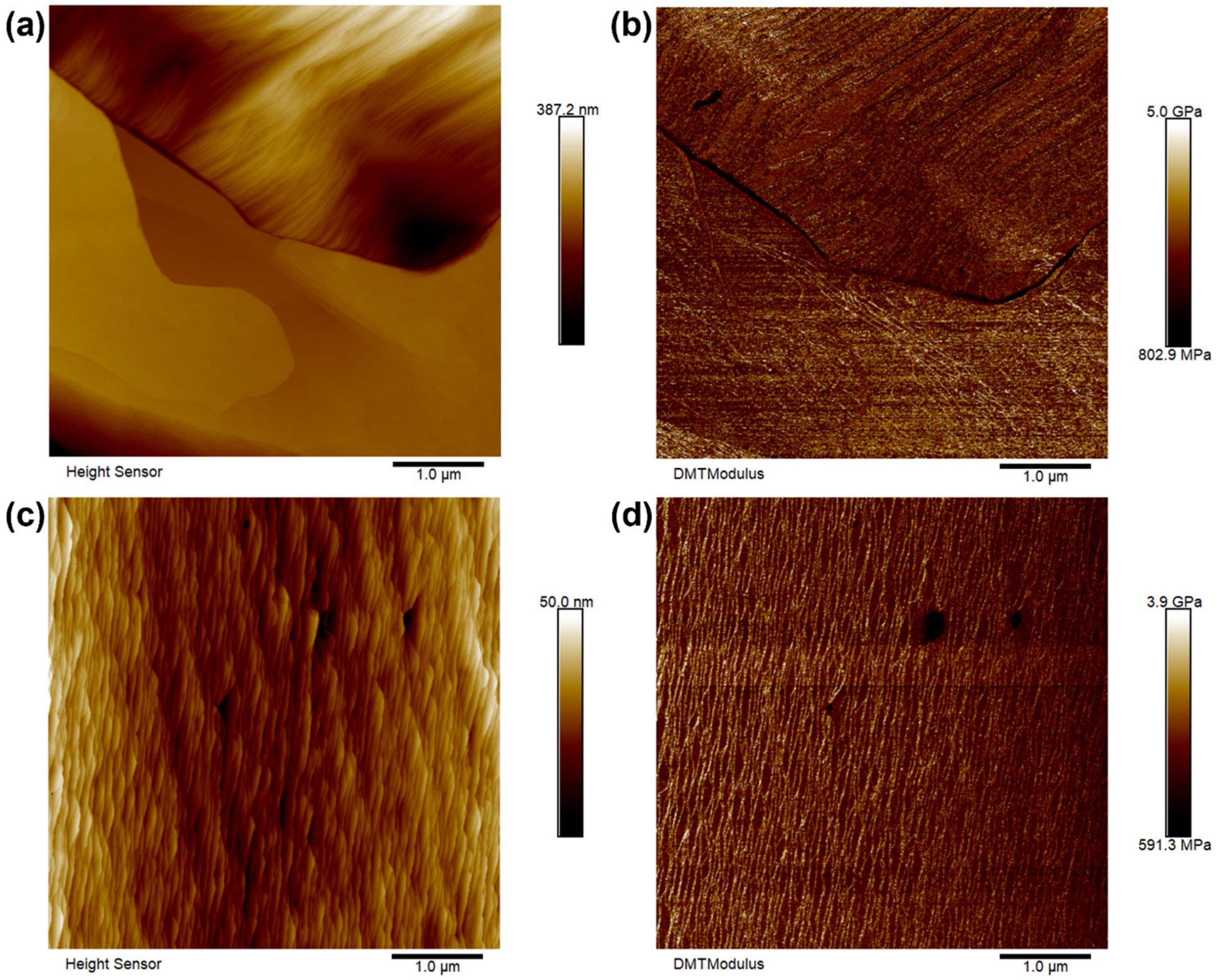

Figure S6. (a) AFM topology image of 2EOImTSA; (b) corresponding AFM modulus image ; (c) AFM topology image of 2EOImTSA at another spot; (d) corresponding AFM modulus image. Figures (a) and (b) were taken on the same spot on the sample, and (c) and (d) were taken from the same spot on the sample.

The AFM images on other spots of the sample also showed great anisotropy. Additionally, the modulus shown in these images (Figure S6b and S6d) also agreed roughly with the rheological measurement results shown in Figure 3.

Dielectric Response of 2EOImTSA

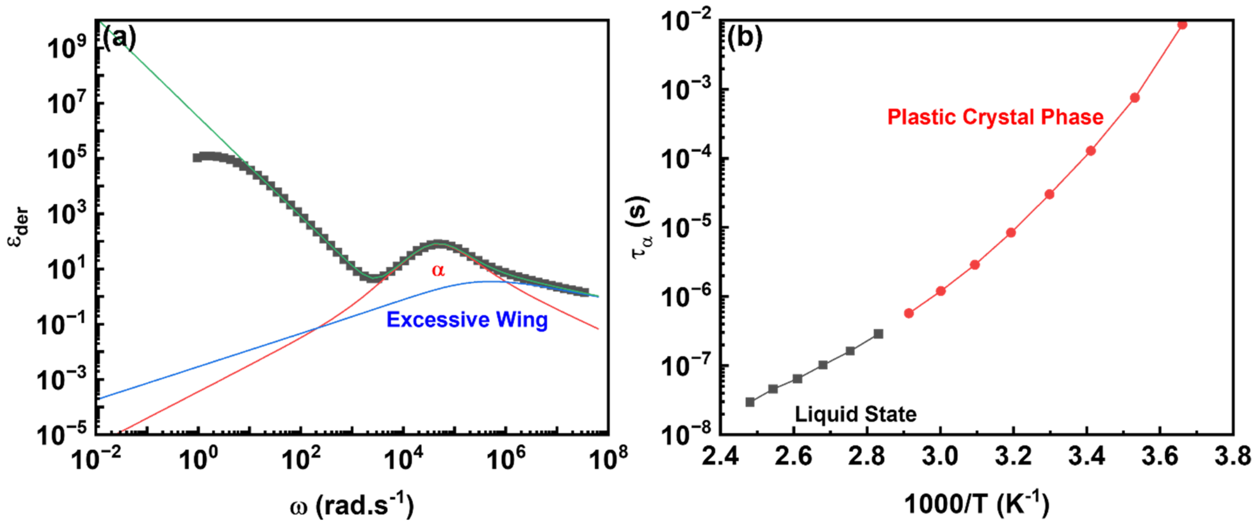

Figure S7. (a) The derivative permittivity at 20 °C calculated from eq. S1, (black squares) as a function of angular frequency. Red and blue lines represent the two fits to the HN equations (eq. S3), and the green line represents the overall fitting line calculated from eq S2; (b) α-relaxation time (τ_α), calculated from eq. S4, as a function of temperature.

To further investigate the dynamics of 2EOImTSA, the relaxation times τ_α are extracted from the derivative permittivity of Figure S7a, calculated as.

$$\varepsilon_{der} = -\frac{\pi}{2}\frac{\partial \varepsilon'(\omega)}{\partial \ln(\omega)} \qquad \text{S1}$$

In which ω is the angular frequency, ε' is the permittivity. ε_{der} is then fitted by the Havriliak–Negami (HN) equations:[4]

$$\varepsilon'' = A\omega^{-s} - \frac{\pi}{2}\left[\frac{\partial \varepsilon'_{HN_1}(\omega)}{\partial \ln(\omega)} + \frac{\partial \varepsilon'_{HN_2}(\omega)}{\partial \ln(\omega)}\right] \qquad \text{S2}$$

$$\varepsilon'_{HN_i}(\omega) = Real\left\{\frac{\Delta\varepsilon_i}{[1+(i\omega/\omega_{HN_i})^{a_i}]^{b_i}}\right\} \qquad \text{S3}$$

in which A and S are constants that control the slope and position of the linear electrode polarization range in the low-frequency regime of the spectra,[5] $\Delta\varepsilon=\varepsilon_s-\varepsilon_\infty$ is the dielectric relaxation strength, a and b are both shape parameters, and ω_{HN} is a characteristic frequency of HN equation.[4] All these constants are fitting parameters. The α-relaxation time (τ_α) can then be calculated as:

$$\tau_\alpha = 2\pi/[\omega_{HN1}(\sin\frac{a_1\pi}{2+2b_1})^{1/a_1}(\sin\frac{a_1b_1\pi}{2+2b_1})^{-1/a_1}] \qquad \text{S4}$$

In the high-frequency regime of the derivative spectra of Figure S7a, a second relaxation process can be seen, which is often denoted as the excessive wing [6](blue line in Figure S7a) and is commonly observed for other PCs. The origin of this high-frequency wing is still uncertain, but many ascribe it to other secondary relaxation processes (such as a β relaxation).[7-9] The temperature dependence of τ_α in the plastic crystal phase, shown in Figure S7b, clearly deviates from an Arrhenius dependence and instead follows a Vogel–Fulcher–Tammann (VFT) [10] temperature dependence, which is also observed for other PCs.[9,11] Therefore, the BDS measurement further proves the existence of the plastic crystal phase in 2EOImTSA, and such properties lead to a high static dielectric constant in a solid crystalline state.

^{1}H and ^{19}F NMR Spectra

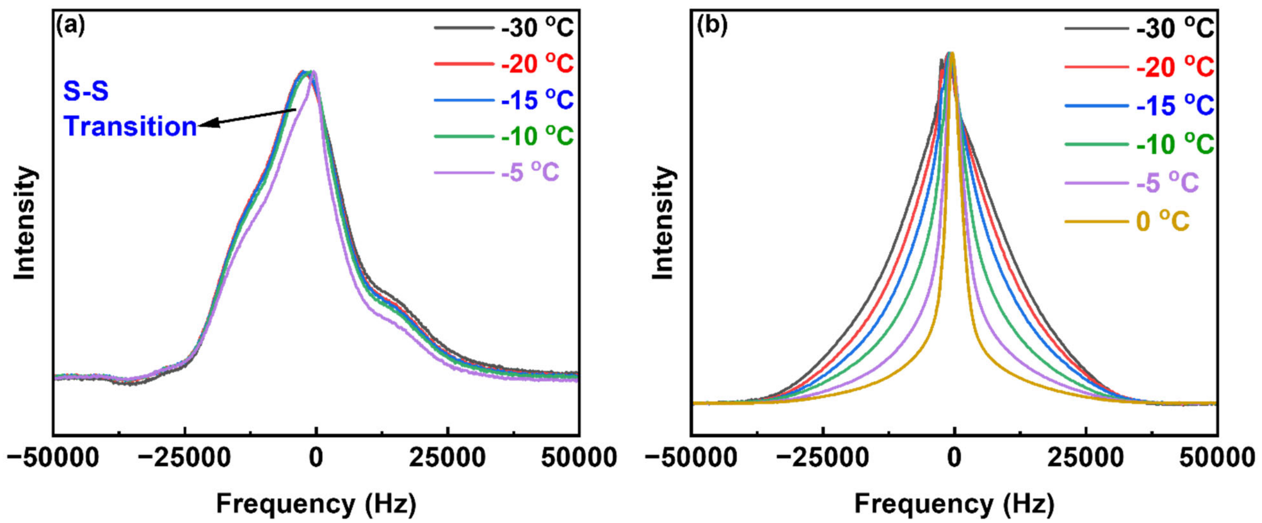

Figure S8. (a) ^{19}F NMR of 2EOImTSA from -30 °C to -5 °C; (b) ^{1}H NMR of 2EOImTSA from -30 °C to 0 °C. The intensity of all spectra was scaled to the maximum intensity of spectra at -30 ℃, respectively for ^{1}H NMR and ^{19}F NMR, for easier comparison.

An abrupt change of peak width was spotted for ^{19}F NMR spectra between -10 °C and -5 °C, which indicates the solid-solid state transition. For ^{1}H NMR spectra, such a change in line width was spotted between -20 °C and -15 °C.

Proton T_1 Relaxation Time

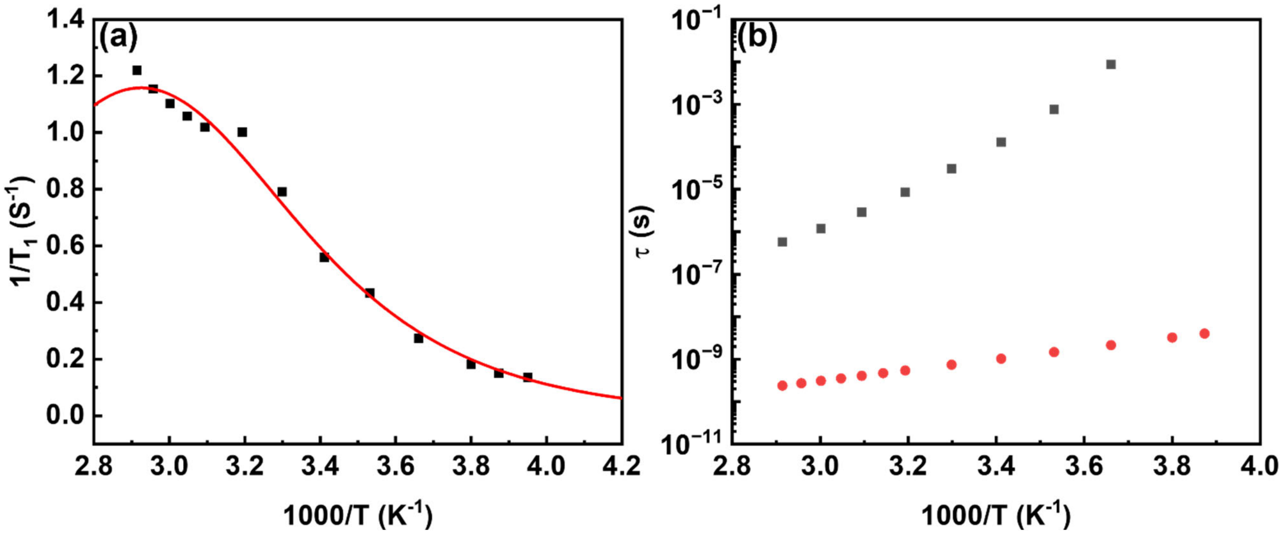

Figure S9. (a) Proton T_1 relaxation time as a function of 1000/T. The red line is the Bloembergen-Purcell-Pound (BPP) fitting curve (eq S5 and S6). (b) τ_c (in red, obtained from fitting data in Figure S9a) and τ_α (in black, obtained from the dielectric relaxation fitting in Figure S7b) as a function of temperature.

The T_1 relaxation time data were fitted using the following equation:

$$\frac{1}{T_1} = A\left[\frac{2\tau_c}{1+\omega_L^2\tau_c^2} + \frac{8\tau_c}{1+4\omega_L^2\tau_c^2}\right] \quad \text{S5}$$

$$\tau_c = \tau_0 \exp\left(\frac{E_a}{k_B T}\right) \quad \text{S6}$$

In which A is a constant, ω_L=2.51×10^9 rad/s is the Larmor frequency, τc is the characteristic relaxation time, which is described using an Arrhenius equation (eq S6). E_a is the activation energy, k_B is the Boltzmann constant, τ_0 is the pre-exponential factor, and T is the temperature. In these equations, A, τ_0, and E_a are fitting parameters. The fitting results are as follows

A=1.0×10^9 s^{-2}; τ_0=4.5×10^{-14} s; E_a=24.4 kJ/mol.

To better quantitatively understand the dynamics of 2EOImTSA in the plastic crystal phase, the proton T_1 relaxation time was measured at various temperatures (Figure S9a), and a characteristic time (τ_c), which represents the dynamics of 2EOImTSA molecules locally, can be obtained from fitting T_1 data using Bloembergen, Purcell, and Pound (BPP) model (Figure S9a).[12] As shown in Figure S9b, this characteristic time (τ_c) is much smaller than the τ_α obtained from BDS measurement (Figure S7b), which represents the time scale of the major dipole relaxation process. Such a discrepancy in relaxation time might indicate that the dipole relaxation process is the result of the collaborative movement of multiple 2EOImTSA molecules. More research is needed to further connect the relaxation times at different length scales.

The Cohesive Energy of Parallel Configuration (++,--) of 2 2EOImTSA Molecules

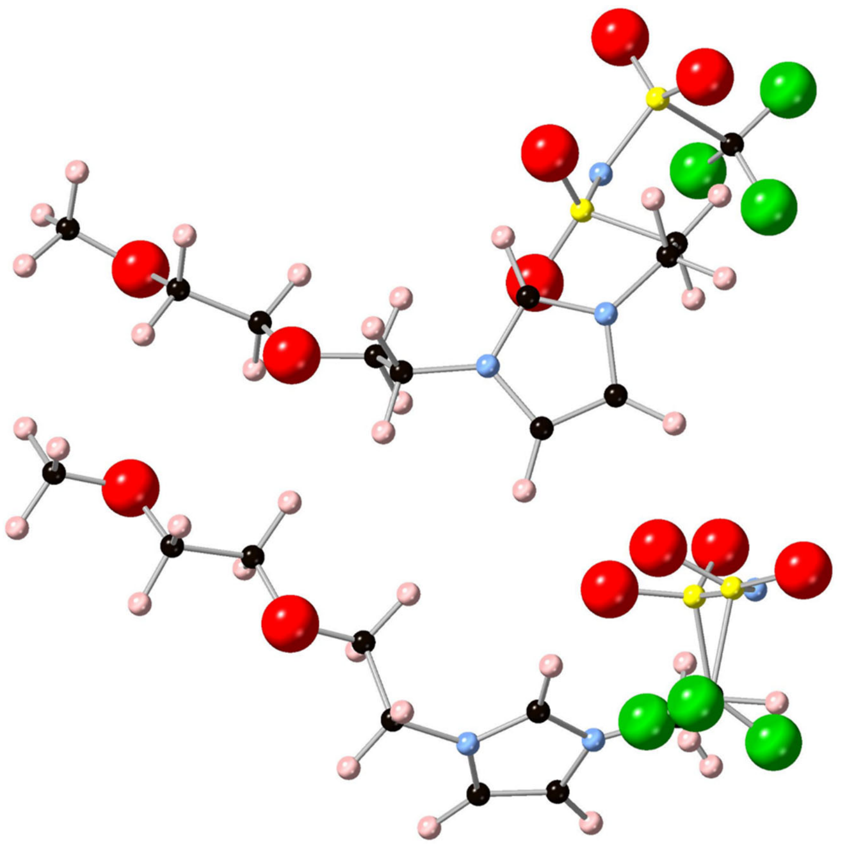

$$\Delta E_{(++,--)} = -117.63\ kJ/molecule$$

Figure S10. Cohesive energy of 2EOImTSA molecules in a parallel conformation calculated using eq1. in the main text. F-green, S-yellow, N-blue, C-black, O-red, H-pink.

The 2EOImTSA ++,-- arrangement exhibited the weakest cohesive energy (−117.63 kJ/mol), primarily due to the large separation between like-charged species, indicating electrostatic repulsion. However, this large separation reduces the magnitude of repulsive interactions. In contrast, the short distance between the anionic and cationic moieties within the same molecule enhances intramolecular attraction. The overall balance between inter- and intramolecular forces

is achieved through structural relaxation, which is facilitated by increased vacuum spacing that allows the chain morphology to adjust and relieve the strain induced by the ++,-- arrangement.

References

1. Dolomanov, O.V., Bourhis, L.J., Gildea, R.J., Howard, J.A.K., and Puschmann, H. (2009). OLEX2: a complete structure solution, refinement and analysis program. Journal of Applied Crystallography 42, 339-341. doi:10.1107/S0021889808042726.
2. Bourhis, L.J., Dolomanov, O.V., Gildea, R.J., Howard, J.A.K., and Puschmann, H. (2015). The anatomy of a comprehensive constrained, restrained refinement program for the modern computing environment - Olex2 dissected. Acta Crystallographica Section A 71, 59-75. doi:10.1107/S2053273314022207.
3. Sheldrick, G. (2015). Crystal structure refinement with SHELXL. Acta Crystallographica Section C 71, 3-8. doi:10.1107/S2053229614024218.
4. Havriliak, S., and Negami, S. (1967). A complex plane representation of dielectric and mechanical relaxation processes in some polymers. Polymer 8, 161-210. https://doi.org/10.1016/0032-3861(67)90021-3.
5. Mei, W., Rothenberger, A.J., Bostwick, J.E., Rinehart, J.M., Hickey, R.J., and Colby, R.H. (2021). Zwitterions Raise the Dielectric Constant of Soft Materials. Physical Review Letters 127, 228001. 10.1103/PhysRevLett.127.228001.
6. Lunkenheimer, P., Michl, M., and Loidl, A. (2018). Nonlinear Dielectric Response of Plastic Crystals. In Nonlinear Dielectric Spectroscopy, R. Richert, ed. (Springer International Publishing), pp. 277-300. 10.1007/978-3-319-77574-6_9.
7. Brand, R., Lunkenheimer, P., Schneider, U., and Loidl, A. (1999). Is There an Excess Wing in the Dielectric Loss of Plastic Crystals? Physical Review Letters 82, 1951-1954. 10.1103/PhysRevLett.82.1951.
8. Brand, R., Lunkenheimer, P., and Loidl, A. (1997). Relaxations and fast dynamics of the plastic crystal cyclo-octanol investigated by broadband dielectric spectroscopy. Physical Review B 56, R5713-R5716. 10.1103/PhysRevB.56.R5713.
9. Brand, R., Lunkenheimer, P., and Loidl, A. (2002). Relaxation dynamics in plastic crystals. The Journal of Chemical Physics 116, 10386-10401. 10.1063/1.1477186.
10. Tammann, G., and Hesse, W. (1926). Die Abhängigkeit der Viscosität von der Temperatur bie unterkühlten Flüssigkeiten. Zeitschrift für anorganische und allgemeine Chemie 156, 245-257. https://doi.org/10.1002/zaac.19261560121.
11. Götz, M., Bauer, T., Lunkenheimer, P., and Loidl, A. (2014). Supercooled-liquid and plastic-crystalline state in succinonitrile-glutaronitrile mixtures. The Journal of Chemical Physics 140. 10.1063/1.4867095.
12. Roach, D.J., Dou, S., Colby, R.H., and Mueller, K.T. (2012). Nuclear magnetic resonance investigation of dynamics in poly(ethylene oxide)-based lithium polyether-ester-sulfonate ionomers. The Journal of Chemical Physics 136. 10.1063/1.3669449.
